# First Identification and Characterization of *Lactococcus garvieae* Isolated from Rainbow Trout (*Oncorhynchus mykiss*) Cultured in Mexico

**DOI:** 10.3390/ani10091609

**Published:** 2020-09-09

**Authors:** Cesar Ortega, Rute Irgang, Benjamín Valladares-Carranza, Constanza Collarte, Ruben Avendaño-Herrera

**Affiliations:** 1 Centro de Investigación y Estudios Avanzados en Salud Animal (CIESA), Facultad de Medicina Veterinaria y Zootecnia (FMVZ), Universidad Autónoma del Estado de México (UAEM), 50295 Toluca, Mexico; bvalladaresc@uaemex.mx; 2 Laboratorio de Patología de Organismos Acuáticos y Biotecnología Acuícola, Facultad de Ciencias de la Vida, Universidad Andrés Bello, 2531015 Viña del Mar, Chile; ruteruben2002@yahoo.ca (R.I.); conyycp@gmail.com (C.C.); 3 Centro FONDAP, Interdisciplinary Center for Aquaculture Research (INCAR), 2531015 Viña del Mar, Chile; 4 Centro de Investigación Marina Quintay (CIMARQ), Universidad Andrés Bello, 2531015 Quintay, Chile

**Keywords:** *Lactococcus garvieae*, rainbow trout, emerging disease, hemorrhagic septicemia lactococcosis, challenge assay

## Abstract

**Simple Summary:**

*Lactococcus garvieae* is an emerging pathogen that causes lactococcosis, which is a septicemia disease that, as per reports, primarily affects rainbow trout and tilapia in various parts of the world. In rainbow trout, the clinical disease is very similar to other diseases caused by Gram-positive bacteria, including weissellosis, which has been previously reported in trout in Mexico. Following septicemia outbreaks that tested negative for weissellosis, a study was conducted to determine the etiology. This study confirms, for the first time, the presence of *L. garvieae* in processes of a septicemia disease affecting five rainbow trout farms in Mexico. Fourteen isolates were obtained from different geographical regions, but presented homogenous biochemical and protein profiles, as well as weak genetic heterogeneity. This study evidences the wide distribution and incidence of lactococcosis cases at rainbow trout farms in Mexico, demonstrating the need to develop vaccination programs against this disease.

**Abstract:**

Lactococcosis is a hyperacute hemorrhagic septicemia disease caused by *Lactococcus garvieae*, which is an emerging pathogen in global fish farming. Between 2016 and 2018, rainbow trout (*Oncorhynchus mykiss*) from five farms that presented outbreaks were sampled as part of a Mexican surveillance program for the detection of fish diseases. Fourteen *L. garvieae* isolates were recovered from sampled fish, as confirmed by biochemical tests, 16S rRNA gene sequencing, and clinical and histological insights. The biochemical and protein profiles of the isolates obtained were homogeneous. Repetitive extragenic palindromic—(REP)—and enterobacterial repetitive intergenic consensus sequence PCR (ERIC-PCR) analyses established weak genetic heterogeneity. Rainbow trout challenged with two of the isolates used at different bacterial concentrations (10^−2^ and 10^−4^ CFU/mL) showed melanosis, and hemorrhages were observed in the fins, liver, kidney, and spleen. Isolates were obtained from all of the organs sampled, including from surviving fish, as either pure or mixed cultures. The present study is the first to confirm the presence of *L. garvieae* as the agent of severe lactococcosis outbreaks in the two primary Mexican states for trout farming.

## 1. Introduction

Lactococcosis is a hyperacute hemorrhagic septicemia disease caused by *Lactococcus garvieae* [1]), which is an emerging pathogen in global fish farming that was initially identified as a cause of bovine mastitis [2]. This microorganism was first reported in 1991 as causing infection in yellowtail (*Seriola quinqueradiata*) farmed in Japan, where it was identified as *Enterococcus seriolicida* [3], and then in rainbow trout (*Oncorhynchus mykiss*) in Australia and Europe [4]. Since then, it has been reported in several parts of the world to affect not only farmed fish, but also shrimp and other fish species from fresh and saline water environments at water temperatures above 15 °C [5,6].

Rainbow trout is one of the most important fish species for aquaculture worldwide, and *L. garvieae* is one of the most critical risks for this industry. Signs in affected fish begin with anorexia, melanosis, and erratic swimming. Other external signs include exophthalmia (uni- or bilateral); hemorrhages in the periorbital and intraocular area, the base of fins, the opercula, and the buccal region; a swollen abdomen; and anal prolapses [7,8].

With regard to aquaculture production in Mexico, the rainbow trout output ranks third in volume (i.e., 14,197 tons, estimated), but second in economic value (CONAPESCA, [9]). Commercial trout production in Mexico has been relatively disease-free since its beginnings in the early 1970s, when eyed eggs were introduced from the United States. Indeed, the only recognized endemic pathogen until 1999 was the infectious pancreatic necrosis virus [10]. However, intensified aquaculture farming since the late 2000s has resulted in the appearance of diseases, such as the recently confirmed rainbow trout fry syndrome (*Flavobacterium psychrophilum*) [11] and weissellosis [12]. The occurrence of infection by Gram-positive microorganisms in rainbow trout has been suspected since 2016, but to date, no cases of streptococcosis or lactococcosis have been officially reported in the country [13].

Between 2016 and 2018, several outbreaks occurred at facilities in the two primary Mexican states for trout farming. Microbiological analyses were conducted as part of a national surveillance program for the detection of fish diseases. Herein, we provide the first report on the isolation, identification, and characterization of *L. garvieae* isolates recovered from associated septicemia processes in Mexican-farmed rainbow trout. These isolates were confirmed by biochemical tests and through 16S rRNA gene sequencing. Clinical and histological insights from the diseased rainbow trout are also provided.

## 2. Materials and Methods

### 2.1. Case Description and Sampling

As part of a Mexican surveillance program for the detection of fish diseases, fish presenting clinical gross signs of hemorrhagic septicemia were sampled from disease outbreaks at different farms between 2016 and 2018 (Table 1). In 2016, commercial-sized rainbow trout (200–300 g) were collected from a farm in the State of Michoacán (farm Mic) and from two farms in the State of Mexico (farms AC and LJ, separated by 6 km). In 2017, rainbow trout (200–500 g) were collected from a single facility (farm RC) in the State of Mexico following symptomatic cases that resulted in morbidity and mortality rates of 50% and 80%, respectively. Finally, rainbow trout (200–400 g) were collected in 2018 from the State of Michoacán at a farm different from that sampled during 2016 (farm LP).

All cases occurred at facilities practicing intensive farming with raceway-type ponds. The following conditions were recorded at all farms: Inflow of spring water exchanged three times daily; an oxygen concentration > 6.5 mg/L; and a temperature between 16 and 18 °C. In all cases, the freshwater is first use water, that is, it has not been previously used by another farm. Five to ten clinically diseased fish (200–500 g) were collected from each outbreak. All fish were transported live in plastic bags with oxygen supplementation to the Aquatic Animal Health Laboratory (Facultad de Medicina Veterinaria y Zootecnia, Universidad Autónoma del Estado de Mexico, Toluca, Mexico) for complete pathological and bacteriological workups. Fish sampling protocols were approved by the Ethics Committee of the Universidad Autónoma del Estado de Mexico under approval accreditation Nº SA-0042-006/12 of the Official Mexican Standard NMX-EC-71025-IMNC-2006 (ISO/IEC 17025: 2005) for conformity assessment activities in Agricultural Health. The Animal Handling Protocol was approved by the Ethics Committee of the Universidad Andrés Bello corresponds to No. 016/2019.

### 2.2. Macroscopic and Histological Analyses

All fish were euthanized via an anesthetic overdose (240 mg/L tricaine methanesulphonate 222; Sigma-Aldrich, Taufkirchen, Germany) and immediately subjected to a post-mortem examination. Analyses were then conducted for the presence of external parasites, and scrapings were obtained from the gills and skin for microscopic observations at 100× and 400× magnifications.

For histological analyses, skin, brain, liver, kidney, heart, and spleen samples were taken from each fish; fixed in vials containing 10% buffered formalin; dehydrated; and embedded in paraffin wax following standard procedures. Each tissue was sectioned at 5 μm and stained with hematoxylin and eosin to describe histopathological alterations, as in Ortega et al. [16]. Sections were observed under a Primo Star Zeiss^®^ microscope (Carl Zeiss AG, Jena, Germany).

### 2.3. Bacteriological Analyses

Slides were also Gram stained and assessed via light microscopy (Primo Star Zeiss^®^ microscope). Additionally, liver, kidney, brain, and external-lesion samples from all assessed fish were streaked onto tryptone soya agar (TSA, Oxoid, Hampshire, UK) supplemented with 1% NaCl (TSA-1) and Columbia sheep blood agar plates (AES Laboratories, Combourg, France) and incubated aerobically at 28 °C for 96 h. Fourteen representative colonies from the different outbreaks were selected from each TSA-1 plate, streaked onto a new TSA-1 plate to obtain pure cultures, and stored at −80 °C in Criobilles tubes (AES Laboratories).

### 2.4. Phenotypic and Biochemical Characterizations

The 14 Mexican isolates obtained from pure bacterial cultures were subjected to the following tests: Gram staining, cytochrome oxidase, catalase, and motility. Growth was also tested on marine agar (BD Difco^TM^, Berkshire, UK), MacConkey agar (Oxoid), nutrient agar (Oxoid), commercial Columbia agar supplemented with 5% defibrinated sheep blood (bioMérieux, Marcy-l’Étoile, France), brain heart infusion agar (Oxoid), Todd-Hewitt agar (Oxoid), thiosulfate-citrate-bile salts-sucrose agar (Oxoid), TSA, TSA-1, and tryptone yeast extracts salts agar [17]. Oxidative/fermentative, arginine dihydrolase, lysine/ornithine decarboxylase, and Simmons citrate reactions were also tested [18]. Additionally, the hydrolysis of gelatine (0.4%, *w*/*v*), starch (0.4%, *w*/*v*), lipase (1% tween 80, *w*/*v*), L-tyrosine (0.4% *w*/*v*), DNAse (Liofilchem, Roseto degli Abruzzi, Italy), and bile esculin (Liofilchem) was tested on TSA agar plates employed as basal medium. Growth at different temperatures was assessed on the TSA growth medium (4, 6, 10, 15, 18, 20, 25, 28, 30, and 37 °C). Different salinities (0–6% NaCl, *w*/*v*) and pH ranges (4–10 pH, 1-unit ranges adjusted with 1N NaOH and HCl) were tested in tryptone soya broth (TSB, BD Difco^TM^). Excepting assessments at different temperatures, all plates and tubes were incubated at 25 °C for 8 d before being scored as negative. The enzymatic profile of each Mexican isolate was defined by employing API ZYM, API 50CH, and rapid ID 32 Strep (bioMérieux), according to the manufacturer’s instructions, except that the temperature was set to 25 °C. The API 50CHL medium was used for assessments with API 50CH. The results of API 50CH and rapid ID 32 Strep were analysed by the input of data in apiweb^TM^ (bioMérieux).

Isolates were also characterized according to Vela et al. [15], where 13 biotypes were proposed based on the acidification of sugars, such as saccharidose, tagatose, mannitol, and cyclodextrin, and on the presence of pyroglutamic acid arylamidase and N-acetyl-β-glucosaminidase enzymes. These characterizations were performed with the rapid ID 32 Strep and API 50CH miniaturized systems, with assay isolates grown on Columbia sheep blood agar for 24 h at 25 °C.

### 2.5. 16S rDNA Sequencing and Phylogenetic Analysis

Genomic DNA was extracted from two pure colonies of 14 isolates, as previously described in Fox et al. [19]. The 16S rRNA gene was PCR-amplified using the sequencing primer pair 27F (5′-AGAGTTTGATCMTGGCTCAG-3′) and 1492R (5′-TACGGYTACCTTGTTACGACTT-3′) [20]. The expected 1500 bp amplicon was obtained and then sequenced by Macrogen (Seoul, Korea). The resulting 16S rRNA sequences were analysed using the Basic Local Alignment Search Tool (BLAST, http://blast.ncbi.nlm.nih.gov/). Type-strain sequences from other species within the same genus were then downloaded, and all of the sequences were aligned using the MEGA v5 software [21]. Genetic distances were obtained using Kimura’s two-parameter model [22] and clustered with the neighbor-joining algorithm. A bootstrap analysis to investigate the stability of the trees was performed on 10,000 replicates.

### 2.6. ERIC-PCR, REP-PCR, and RAPD-PCR Typing

To analyse the intraspecific genetic variability between the 14 rainbow trout isolates, enterobacterial repetitive intergenic consensus sequence PCR (ERIC-PCR), repetitive extragenic palindromic PCR (REP-PCR), and randomly amplified polymorphic DNA (RAPD) assays were employed. These PCR analyses are fast, simple, and frequently used for the genomic fingerprinting of fish and shellfish pathogens [23]), including *Lactococcus* species [24,25].

RAPD amplifications were performed using Ready-To-Go^TM^ (Sigma-Aldrich) RAPD analysis beads with Primer 5 (5′-d[AACGCGCAAC]-3′), as recommended by Ravelo et al. [24]. In turn, the REP-PCR and ERIC-PCR amplifications were performed using the commercial kit GoTaq^®^ Green Master Mix (Promega, Madison, WI, USA). All PCRs were carried out in a G-Storm T Gradient Thermal Cycler (Akribis Scientific Limited, Cheshire, UK). Specific assay primers and amplification protocols were as previously described in Mancuso et al. [26]. PCR products were separated by 2% agarose gel electrophoresis and stained with the SafeView Nucleic Acid Stain (NBS Biologicals, Cambridgeshire, UK). All runs included a negative control, which used distilled water instead of template DNA. The RAPD assay also included a positive control, which used *Escherichia coli* BL21 DNA (included in the kit). The gels were photographed under UV light using the AccuRuler 100 bp Plus DNA RTU Ladder (Maestrogen, Hsinchu, Taiwan) as a molecular-mass marker. Data analyses were performed using the GelJ software version 3.0 (Java Application, Logroño, Spain), and the computed similarities among strains were estimated by means of the Dice coefficient [27]. A dendrogram was produced based on the unweighted average pair group method.

### 2.7. Membrane Protein Characterization

Whole-cell proteins from each isolate were prepared from bacterial cultures grown in TSB for 48 h at 25 °C with agitation. Then, the bacteria were centrifuged at 8000× *g* for 10 min at 4 °C. The resulting pellet was suspended in PBS (pH 7.4) at a concentration of 10^9^ CFU mL^−1^, washed once in the same saline solution by centrifugation at 8000× *g* for 3 min at 4 °C, and stored at −20 °C until required.

The total membrane protein was obtained using the method described by Crosa and Hodges [28]. The pellet was resuspended in 2 mL of 10 mM Tris (hydroxymethyl)aminomethane (Tris)-hydrochloride with 0.3% NaCl (pH 8.0) and sonically treated by four 15 s cycles, with maintenance for 4 s on ice between the sonication cycles. Samples were pelleted by centrifugation at 10,000× *g* for 2 min at 4 °C, and supernatants were transferred into new collector tubes prior to determining protein concentrations. The protein concentration was quantified using the Pierce^TM^ BCA Protein Assay Kit (Thermo Fisher Scientific), and samples (25 mg mL^−1^) were electrophoresed by SDS-PAGE using 15% acrylamide-separating gel with 5% stacking gel [29]. After electrophoresis (80 V for 10 min and then 120 V for 90 min), protein bands were stained with Coomassie brilliant Blue R-250 Dye in 50% (*v*/*v*) methanol and 10% (*v*/*v*) acetic acid. Gels were de-stained with 10% acetic acid and 40% methanol, and protein profiles were analysed using the Image Lab v6.0 software package (Bio-Rad, Hercules, CA, USA).

### 2.8. Fulfilment of Koch’s Postulates

Challenge assays used healthy pathogen-free rainbow trout averaging 10.5 g in weight and 11 cm in size. These fish were obtained from a fish farm with no history of disease. Fish were fed daily (1% body weight) with commercial pellets; dechlorinated water was changed every other day, and the light:dark regime was 12:12 h. Fish were kept in aerated conical tanks (200 L) until the start of the trial, at which point, fish were grouped into 8 fish per 8 L aquarium. The water temperature was kept at 17 ± 1 °C throughout the experimental period.

Two Mexican isolates were employed in the challenge assays (encoded RC17-Lg06 and Mic16-Lg07). Mic16-Lg07 was chosen as it was one of the stored isolates from 2016, and RC17-Lg06 was chosen as it was recovered from a 2017 outbreak. For the trials, the Mic16-Lg07 and RC17-Lg06 isolates were incubated in TSB for 24 h at 18 °C with initial respective concentrations of 7.4 × 10^8^ and 1.9 × 10^9^ CFU per mL, as quantified by serial dilution. Virulence tests were performed by intraperitoneal injections at two dilutions for each isolate—10^−2^ and 10^−4^ CFU. Control fish were injected with TSB without bacteria. Another tank with fish without injection treatment was included as a control and for the monitoring of fish. Both the experimental and control treatments were tested in duplicate. The challenge period lasted up to 12 d, during which time, each group was observed daily for clinical signs. Any dead fish were removed from the tank and sampled by TSA-streaking skin lesions and/or hemorrhages (if present); exophthalmia (if observed); and spleen, liver, kidney, and brain tissues.

If pure colonies were obtained, biochemical and PCR methods were used for identification. For this, DNA was extracted from the representative colonies grown on agar plates and subjected to PCR analysis using the specific primers pLG-1 (5′-CATAACAATGAGAATCGC-3′) and pLG-2 (5′-GCACCCTCGCGGGTTG-3′) [30]) and the commercial kit GoTaq^®^ G2 DNA Polymerase (Promega), which included all reaction reagents, except for the specific primer and DNA template. Negative controls used the same reaction mixture and sterile distilled water. The products were visualized with a 1/10,000 GelRed Nucleic Acid Gel Stain (Biotium, Fremont, CA, USA) and photographed under UV light. An AccuRuler 100 bp Plus DNA RTU Ladder (Maestrogen) was used as a molecular mass marker. A single 1100 bp product was considered as positive for *L. garvieae*.

## 3. Results and Discussion

### 3.1. Macroscopic, Histological, and Bacteriological Analyses

**Figure 1:**
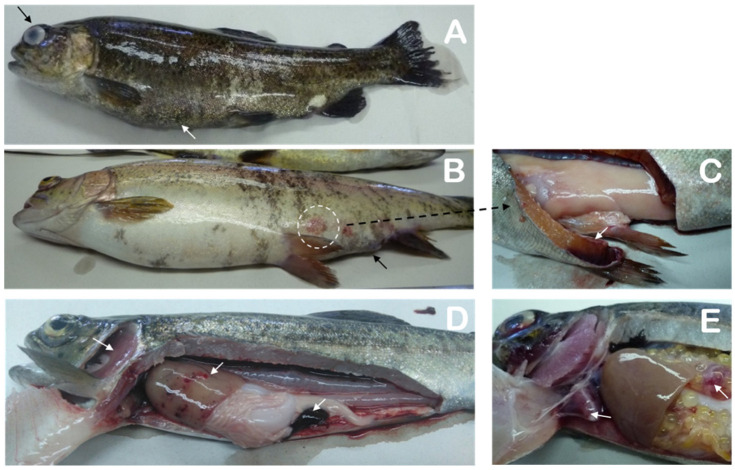
Rainbow trout with *Lactococcus garvieae* lesions. Shown are (**A**) melanosis and abdominal bulging (white arrow), as well as exophthalmia and corneal opacity (black arrow); (**B**) hemorrhaging on the base of the fins, anal prolapse (black arrow), hemorrhages on the skin, and rounded muscle bulges (2–3 cm diameter) on the skin (circle) and (**C**) in the deep-muscle layers. Necropsies revealed (**D**) generalized pallor, hemorrhages in the liver and intestine, and splenomegaly (white arrows), as well as (**E**) fibrinous adhesions in the heart (arrow) and hemorrhages in the ovary (arrow).

All of the fish sampled were selected based on their abnormal behavior (e.g., anorexia, lethargy, loss of orientation, and erratic swimming) and macroscopic clinical signs (e.g., melanosis, abdominal bulging, exophthalmia, and corneal opacity) (Figure 1A). Some fish presented hemorrhaging on the base of the fins and in different parts of the body, as well as suffered an anal prolapse (Figure 1B). All of these clinical signs are presumptively consistent with *L. garvieae* infection [5,6]. Likewise, microscopic examinations of wet-mount smears from these lesions revealed the presence of high quantities of cocci in short chains and pairs of bacteria, while Gram-staining indicated that the bacteria were Gram-positive. Interestingly, three rainbow trout sampled in 2017 presented rounded muscle bulges (2–3 cm diameter) with severe hemorrhaging in the skin and deep-muscle layers (Figure 1B,C)—lesions not previously reported in cases of lactococcosis in fish. Macroscopic necropsies detected severe anemia, pale gills, congestion and hemorrhages of the liver and intestine, and splenomegaly (Figure 1D). Some fish had ascites, fibrinous adhesions in the heart and towards the pleura, hemorrhaging in the ovary (Figure 1E), congestion and hemorrhages in the swim bladder, and renomegaly (data not shown).

**Figure 2:**
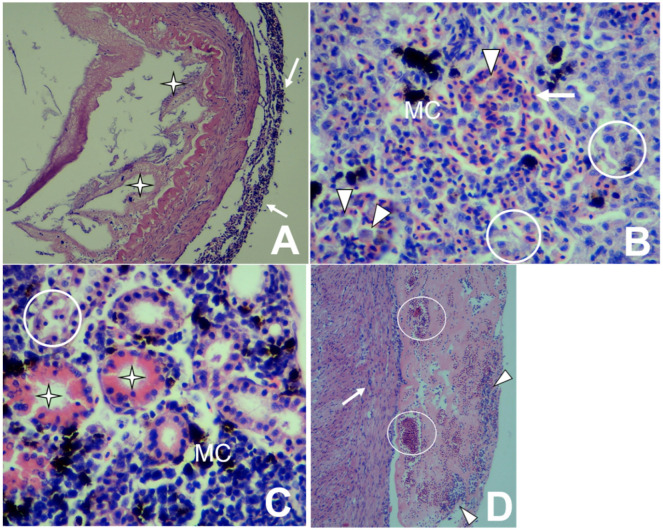
Histopathological changes in rainbow trout infected with *Lactococcus garvieae*. (**A**) Intestine: Mononuclear peritonitis (arrows) with a loss of intestinal mucosa and villi (stars) [H&E stain 100×]. (**B**) Liver: Mononuclear hepatitis with congestion (arrow), degeneration and necrosis (circles), macrophage proliferation (arrowheads), and melano-macrophage centers (MC) [H&E stain 400×]. (**C**) Kidney: Degeneration and tubular necrosis (circle) with the presence of proteinaceous material within tubules (star) and the proliferation of melano-macrophage centers (MC) [H&E stain 400×]. (**D**) Heart: Mononuclear epicarditis (arrow) and pericarditis (arrowheads), as well as congestion (circles) [H&E stain 100×].

*Lactococcus garvieae* causes vascular endothelial lesions leading to the manifestation of petechiae and disseminated hemorrhaging [5,6]. In the present study case, the histological lesions confirmed septicemia manifestation. Histologically, disseminated bacterial forms were found in the liver, kidney, and spleen (data not shown). These were accompanied by necrotizing peritonitis (Figure 2A); multifocal mononuclear hepatitis; liver congestion and bleeding (Figure 2B); tubular degeneration (Figure 2C); mononuclear pericarditis and epicarditis with thrombus formation (Figure 2D); in the spleen, necrotizing splenitis; and brains with mononuclear meningitis and encephalitis, congestion, and cerebral hemorrhages (data not shown).

### 3.2. Bacteriological and Biochemical Analyses

From the sampled fish, only 14 representative isolates were stored for posterior identification: Six were obtained in 2016 (two from the State of Mexico and four from the State of Michoacán); 6 in 2017; and 2 in 2018 (Table 1). Phenotypic and biochemical test results showed an acceptable match for the identification of each isolate as *L. garvieae* [5,14,15], with a great degree of homogeneity observed among the Mexican isolates, regardless of the year and geographical area. Colonies, regardless of the culture media, were similarly circular and smooth and had no pigmentation. Isolates grew on all of the tested culture media except thiosulfate-citrate-bile salts-sucrose and MacConkey agar. Growth of the Mexican isolates on blood agar was tested at different temperatures, and no hemolysis was recorded at any temperature tested (i.e., 18–37 °C), even after 40 d of incubation. Cells were Gram-positive ovoid cocci that were non-motile and could be observed alone, forming short chains. Catalase and oxidase reactions were negative. Slant growth was observed at pH 8–9, but turbidity was still observed when compared to non-inoculated wells. For the API ZYM reactions, all isolates were negative for alkaline phosphatase, esterase (C4), lipase (C14), valine and cystine arylamidase, trypsin, α- and β-galactosidase, β-glucuronidase, N-acetyl-β-glucosaminidase, α-mannosidase, and α-fucosidase. No differences were found for the API 50CH reactions after 48–96 h of incubation. Positive results were also observed for D-galactose, D-glucose, D-fructose, D-cellibiose, and D-trehalose, and weekly positive results were found for N-acetylglucosamine, amygdaline, salicin, and gentiobiose. Regarding the rapid 32ID Strep mini gallery, positive reactions were observed for L-arginine and the acidification of β-glucoside, D-ribose, D-trehalose, D-raffinose, D-maltose, and methyl-βD-glucopyranosidase. The remaining results were negative, regardless of the strips tested, with full details given in Table 1. Moreover, the 14 isolates rendered positive reactions for saccharidose, tagatose, mannitol, cyclodextrine, and the presence of pyroglutamid acid arylamidase and N-acetyl-β-glucosaminidase enzymes. These findings would classify the 14 isolates as biotype 2, according to the scheme of Vela et al. [15], which is a study that classified Spanish trout isolates as biotype 2. The apiweb results rendered all 14 Mexican isolates as *L. garvieae,* with a 99.7% (3036 3101 131) identification profile expected for this bacterial species.

### 3.3. 16S rDNA Sequencing and Phylogenetic Analysis

**Figure 3:**
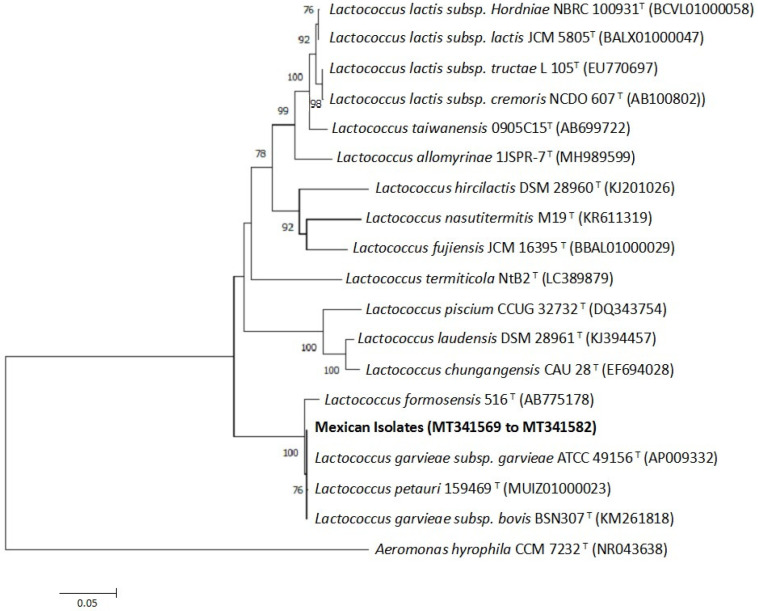
Inferred species relationships between the 14 Mexican isolates and other *Lactococcus* species, based on a neighbor-joining analysis of partial 16S rRNA gene sequences (1401 bp). Numbers at the nodes are bootstrap values (% of 10,000 replications; only values > 70% are shown). Bars indicate 0.05 substitutions per nucleotide.

Sequencing analysis of the nearly complete (i.e., 1401 bp) 16S rDNA gene revealed that all of the Mexican isolates studied were identical, with the obtained sequence showing 100% similarity with *L. garvieae* subsp. *garvieae* ATCC 49156^T^ (GenBank accession number AP009332) (Figure 3). The next nearest species were *Lactococcus petauri* 159469^T^ (accession number MUIZ01000023) and *Lactococcus garvieae* subsp. *bovis* BSN307T (accession number KM261818), with similarities of 99.9% and 99.6%, respectively. The 14 16S rRNA nucleotide sequences were deposited in GenBank (Accession numbers MT341569 to MT341582).

### 3.4. ERIC-PCR, REP-PCR, and RAPD-PCR Typing

PCR-based typing is an effective approach in the epidemiological study of *L. garvieae* strains [23,24]. These studies have supported the existence of genetic variability among isolates, which was associated with the geographical origin, but not entirely with the ecological niche of origin, for *L. garvieae*. In the present study, differences were observed between the Mexican isolates according to the typing method employed. Aside from some variations in the band intensity, no differences were observed between the profiles obtained for each Mexican isolate, and all of the banding patterns were highly reproducible with every PCR tool.

**Figure 4:**
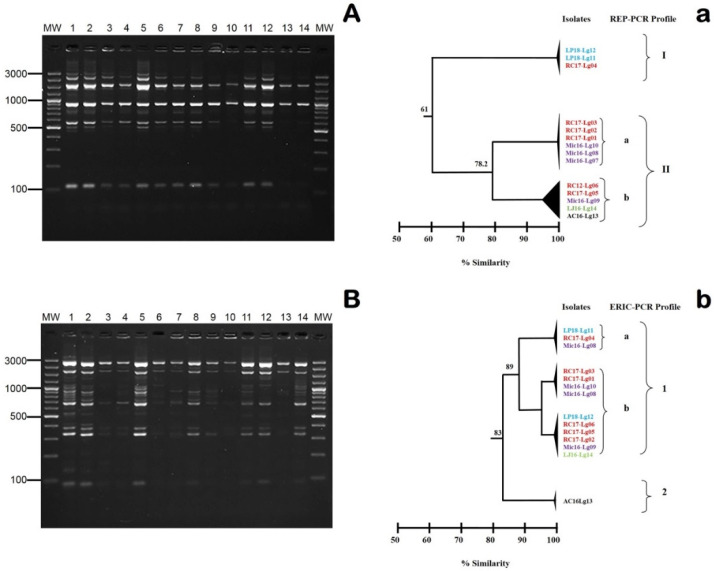
(**A**) Repetitive extragenic palindromic PCR (REP-PCR) and (**B**) enterobacterial repetitive intergenic consensus sequence PCR (ERIC-PCR) fingerprinting obtained for the 14 Mexican *L. garvieae* isolates. Lanes: MW, AccuRuler 100 bp Plus DNA RTU Ladder (Maestrogen); 1, AC16-Lg13; 2, LJ16-Lg14; 3, Mic16-Lg07; 4, Mic16-Lg08; 5, Mic16-Lg09; 6, Mic-Lg10; 7, RC17-Lg01; 8, RC17-Lg02; 9, RC17-Lg03; 10, RC17-Lg04; 11, RC17-Lg05; 12, RC-Lg06; 13, LP18-Lg11; and 14, LP18-Lg12 correspond to the numerical code of DNA extracted from each isolate. Numbers on the left indicate the position of the molecular-size marker in bp. Dendrograms were established by the GelJ software (Java Application) using the Dice similarity coefficient and the unweighted average pair group method on the basis of the (**a**) REP and (**b**) ERIC profiles. Codes in the same colors indicate isolates obtained from the same Mexican farm. Numbers along the branches indicate % similarity.

When RAPD fingerprinting using Primer 5 was conducted, only one genetic profile was observed for the Mexican isolates, regardless of the isolation year and farm (data not shown). However, the patterns obtained with REP-PCR and ERIC-PCR differentiated two genetic groups within the 14 Mexican isolates, with approximate similarity levels of 61% (I and II) and 83% (1 and II), respectively (Figure 4). In both cases, two subgroups were detected, with similarity levels of 78.2% and 89% for REP-PCR (Figure 4A) and ERIC-PCR (Figure 4B), respectively. Despite *L. garvieae* LP18-Lg11 and RC17-Lg04 yielding specific profiles (i.e., grouping into a minority cluster, regardless of the technique used), no epidemiological association could be established between the studied isolates. In relation to this point, none of the evaluated farms (even those in proximity to one another) shared water resources or any recent inflow of clinically ill fish. Therefore, future studies with more isolates should explore the origin of the *L. garvieae* infections.

### 3.5. Membrane Protein Analyses

**Figure 5:**
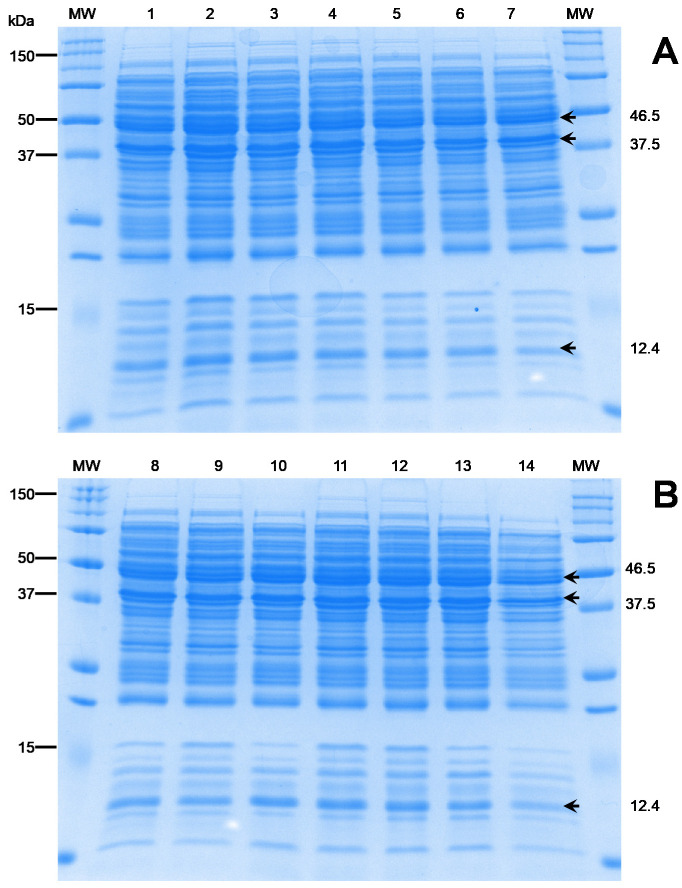
Electrophoresis protein profiles of 14 Mexican *L. garvieae* isolates. Lanes: MW, BioRad Precision Plus Protein^TM^; (**A**) (1–7): 1, RC17-Lg01; 2, RC17-Lg02; 3, RC17-Lg03; 4, RC17-Lg04; 5, RC17-Lg05; 6, RC-Lg06; and 7, Mic16-Lg07 isolates. (**B**) (8–14): 8, Mic16-Lg08; 9, Mic16-Lg09; 10, Mic-Lg10; 11, LP18-Lg11; 12, LP18-Lg12; 13, AC16-Lg13; and 14, LJ16-Lg14. Numbers on the left indicate positions in the molecular size marker (kDa). Numbers on the right indicate the molecular size of differential bands.

Cell-wall proteins are targets of immune surveillance and thereby contribute to serological differences between strains, which is used to guide the focus of vaccine-development studies [6]. As an initial step towards this larger aim, the present study evaluated the existence of protein-profile variability among the Mexican isolates recovered from different farms. The results clearly indicated a unique identical pattern between the 14 Mexican isolates (Figure 5). Protein bands with approximate molecular masses of between 10 and 150 kDa were observed. This finding aligns with those from other studies on *L. garvieae* [31]. It is important to note that a considerable number of more intense bands are given between 10 and 50 kDa, where those with molecular masses of around 12.4, 37.5, and 47.5 kDa stand out. These band observations are also consistent with previous antigenic protein studies carried out for the development of vaccines against *L. garvieae* [31,32,33]. These findings suggest that the Mexican isolates are a homogenous species, regardless of the isolation year and geographical area.

### 3.6. Challenge Assays

The first mortalities were recorded at 48 h after the start of the challenge assay for both isolates at a 10^−2^ dilution (12.5%). The gross mortality (56%) significantly increased after the first 72 h, as did the number of fish affected by either isolate/dilution. After 72 h, the number of deceased fish doubled for the RC17-Lg06 isolate at a 10^−4^ dilution compared to the Mic16-Lg07 isolate at a 10^−4^ dilution. On day 4, all fish challenged at a 10^-2^ dilution had died (*n* = 32; 8 fish per tank in duplicate), regardless of the isolate tested. For the 10^-4^ dilutions, overall survival rates were 43% for the Mic16-Lg07 isolate and 12.5% for the RC17-Lg06 isolate. No mortalities were recorded on days 6 and 7 for fish challenged at a 10^−4^ dilution against RC17-Lg06 and Mic16-Lg07, respectively. Control fish did not present any mortalities or changes in health status during the entire challenge period. At day 12 after the challenge start, all surviving fish were euthanized with an overdose of benzocaine. These fish were analysed as possible carriers of *L. garvieae,* despite feeding normally and not presenting changes in their health status.

The first external changes to health included skin darkening (prevalently observed at a 10^−2^ dilution for both isolates) and scale loss (commonly observed at 10^−4^ dilutions, but most fish maintained healthy skin). Hemorrhagic fins were a usual aspect observed with the 10^−2^ dilution. While most 10^−4^/Mic16-Lg07 fish presented no changes on the fins, more fish challenged with the RC17-Lg06 isolate (50%) were affected, regardless of the dilution employed. Considering all challenged fish, most eye alterations were recorded at the 10^−2^ dilution (≈60% darkened eyes; 9% exophthalmia; and 31% without changes). In contrast, no significant eye changes were observed at the 10^−4^ dilution. Internally, half of the fish challenged with the 10^−2^ dilution presented a pale liver, and another half of the 10^−4^ dilution fish showed a hemorrhagic liver. Both of these injuries were observed, regardless of the isolate. The same number of elongated spleens was found for the 10^−2^ and 10^−4^ dilutions of the isolate Mic16-Lg07. For the RC17-Lg06 isolate, the same number of specimens showed splenomegaly at both dilutions. Friable kidneys were a usual organ problem observed for both isolates at a 10^−4^ dilution and also at a 10^−2^ dilution for the isolate Mic16-Lg07. Kidney inflammation was frequently observed for the RC17-Lg06 isolate at a 10^−2^ dilution. The challenge isolates did not heavily affect the brain, with only 12.5% of all fish specimens presenting hemorrhagic brains. Control and surviving fish presented healthy skin, fins, eyes, livers, and spleens.

Pure cultures of the *L. garvieae* isolates were obtained mostly from the brain (65%) and spleen (64%), regardless of the isolate or dilution. Isolates were recovered from liver (64%) and kidney (80%) samples, mostly in mixed cultures. Both isolates and dilutions were recovered from all sampled fish and organs, except from one surviving fish that yielded no growth. Bacterial colonies similar to *L. garvieae* were observed at a significantly lower number (<10 CFU/sample) on plates cultured from surviving fish compared to plates cultured from dead fish. All isolates obtained from dead and euthanized fish were confirmed as *L. garvieae* through biochemical and PCR analyses (data not shown). No bacterial colonies were recorded for the control specimens. These findings indicate that disease is reproduced in rainbow trout, clearly supporting the high pathogenic potential of the *L. garvieae* isolates for Mexican *O. mykiss* aquaculture.

To date, *L. garvieae* has been isolated from *O. mykiss* in Africa, Oceania, Europe, and Asia [6]. While a genome description exists of an *L. garvieae* isolate obtained from an outbreak in North America [34], our results significantly expand the geographical distribution of *L. garvieae* to include rainbow trout farming on the American continent, with several reported outbreaks occurring between 2016 and 2018. Moreover, it is probable that this pathogen is present in other countries in America, but there are no records in the scientific literature.

The origin of *L. garvieae* in the five farms and the possible route of infection in rainbow trout remain to be clarified. Nevertheless, water temperatures above 16 °C have been attributed as a main trigger for lactococcosis outbreaks [35].

## 4. Conclusions

The present study is the first to confirm the presence of *L. garvieae* as the agent of severe outbreaks in two of the main states in Mexico for trout farming. The assessed isolates obtained from the outbreak were homogeneous biochemically and in terms of the protein profile. Similarly, weak genetic heterogeneity was established by REP- and ERIC-PCR analyses. Until now, these findings suggest that the Mexican isolates are a homogenous species, regardless of the isolation year and geographical area.

## Figures and Tables

**Table 1:** Results obtained for the 14 Mexican rainbow trout isolates compared to the *Lactococcus garvieae*. Data were obtained in this study unless elsewhere mentioned. +, positive; −, negative; F, fermentation; w, weakly positive; v, variable results between API 50CH and rapid ID32 Strep; nd, not determined; * A/L/O, arginine dihydrolase and lysine and ornithine decarboxylase; 1, data obtained from Vendrell et al. [5]; 2, Ravelo et al. [14]; 3, Vela et al. [15]; β-NAG, n-acetyl- β-glucosaminidase; APPA, alanine phenylanaline proline arylamidase; GTA, glycyl tryptophan arylamidase; PYRA, pyrrolidonyl arylamidase.

Characteristics	AC16-Lg13	LJ16-Lg14	Mic16-Lg07	Mic16-Lg08	Mic16-Lg09	Mic16-Lg10	RC17-Lg01	RC17-Lg02	RC17-Lg03	RC17-Lg04	RC17-Lg05	RC17-Lg06	LP18-Lg11	LP18-Lg12	*L. garvieae* ^1^
State	Mexico	Mexico	Michoacán	Michoacán	Michoacán	Michoacán	Mexico	Mexico	Mexico	Mexico	Mexico	Mexico	Michoacán	Michoacán	Review
Isolation (month/year)	07/2016	07/2016	12/2016	12/2016	12/2016	12/2016	12/2017	12/2017	12/2017	12/2017	12/2017	12/2017	08/2018	08/2018	Review
Farm	AC	LJ	Mic	Mic	Mic	Mic	RC	RC	RC	RC	RC	RC	LP	LP	nd
Fish affected (g)	200–300	200-300	200–300	200–300	200–300	200–500	200–500	200–500	200–500	200–500	200–500	200–500	200–400	200–400	−
Nº of fish sampled	5	8	8	8	8	8	10	10	10	10	10	10	8	8	nd
Density (kg/m^3^)	15	14	15	15	15	17	17	17	17	17	17	17	20	20	−
Source	Brain	Heart	Kidney	Liver	Kidney	Heart	Liver	Spleen	Heart	Brain	Kidney	Kidney	Kidney	Brain	−
Gram	+	+	+	+	+	+	+	+	+	+	+	+	+	+	+
Morphology	Ovoid	Ovoid	Ovoid	Ovoid	Ovoid	Ovoid	Ovoid	Ovoid	Ovoid	Ovoid	Ovoid	Ovoid	Ovoid	Ovoid	Ovoid
Motility	−	−	−	−	−	−	−	−	−	−	−	−	−	−	−
Catalase	−	−	−	−	−	−	−	−	−	−	−	−	−	−	−
Oxidase	−	−	−	−	−	−	−	−	−	−	−	−	−	−	−
O/F	F	F	F	F	F	F	F	F	F	F	F	F	F	F	F
Citrate	−	−	−	−	−	−	−	−	−	−	−	−	−	−	−
DNAsa	−	−	−	−	−	−	−	−	−	−	−	−	−	−	nd
Vogues-Proskauer	+	+	+	+	+	+	+	+	+	+	+	+	+	+	+
A/ L/O *	+/−/−	+/−/−	+/−/−	+/−/−	+/−/−	+/−/−	+/−/−	+/−/−	+/−/−	+/−/−	+/−/−	+/−/−	+/−/−	+/−/−	+/−/−
Hydrolisys															
Starch	−	−	−	−		−	−	−	−	−	−	−	−	−	−
Casein	−	−	−	−	−	−	−	−	−	−	−	−	−	−	nd
Esculin	+	+	+	+	+	+	+	+	+	+	+	+	+	+	+
Gelatine	−	−	−	−	−	−	−	−	−	−	−	−	−	−	nd
Tween 80	−	−	−	−	−	−	−	−	−	−	−	−	−	−	nd
API galleries															
Esterase lipase	+	+	+	−	+	−	+	+	+	+	−	−	−	−	nd
Leucine arylam	+	+	+	+	+	+	+	+	+	+	+	+	+	+	+
α-chemotrypsin	−	−	+	−	+	−	+	+	+	−	−	+	−	−	nd
α-glucosidase	+	+	+	+	+	+	−	−	−	+	−	−	+	+	nd
β-galactosidase	−	−	−	−	−	−	−	−	−	−	−	−	−	−	v^2^
β-glucosidase	+	+	+	+	+	+	−	−	−	+	−	−	+	+	nd
β-glucuronidase	−	−	−	−	−	−	−	−	−	−	−	−	−	−	−^2^
β-NAG	−	−	−	−	−	−	−	−	−	−	−	−	−	−	v^3^
D-maltose	+	+	+	+	+	+	+	+	+	+	+	+	+	+	+
D-mannitol	+	+	+	+	+	+	+	+	+	+	+	+	+	+	+
D-mannose	+	+	+	+	+	+	+	+	+	+	+	+	+	+	+
D-melezitose	−	−	−	−	−	−	−	−	−	−	−	−	−	−	−
D-raffinose	v	v	v	v	v	v	+	−	v	v	v	v	v	v	−
D-ribose	+	+	+	+	+	+	+	+	+	+	+	+	+	+	v
D-sorbitol	−	−	−	−	−	−	−	−	−	−	−	−	−	−	+
D-tagatose	+	+	+	+	+	+	+	+	+	+	+	+	+	+	v
L-arabinose	−	−	−	−	−	−	−	−	−	−	−	−	−	−	−
APPA	+	+	+	+	+	+	+	+	+	+	+	+	+	+	+^2^
Cyclodextrin	+	+	+	+	+	+	+	+	+	+	+	+	+	+	v^3^
Glycogen	−	−	−	−	−	−	−	−	−	−	−	−	−	−	−
GTA	−	−	−	−	−	−	+	−	−	−	−	−	−	−	−^2^
Lactose	−	−	−	−	−	−	−	−	v	−	v	−	−	−	w
PYRA	+	+	+	+	+	+	+	+	+	+	+	+	+	+	v
Sucrose	+	+	+	+	+	+	+	+	+	+	+	+	+	+	v
Urease	−	−	−	−	−	−	−	−	−	−	−	−	−	−	−
Temperature (°C)	5–37	5–37	5–37	5–37	5–37	5–37	5–37	5–37	5–37	5–37	5–37	5–37	5–37	5–37	4–45
Salinities (%)	0–9	0–9	0–9	0–8	0–8	0–8	0–7	0–8	0–7	0–7	0–9	0–9	0–8	0–8	0–6.5
pH range	5–10	5–10	5–10	5–10	5–10	5–10	5–10	5–10	5–10	5–10	5–10	5–10	5–10	5–10	7–9.6
Biotype	2	2	2	2	2	2	2	2	2	2	2	2	2	2	v

## References

[1] Austin B., Austin A.D. (2012). Bacterial Fish Pathogens: Diseases of Farmed and Wild Fish.

[2] Collins M.D., Farrow J.A., Phillips B.A., Kandler O. (1983). *Streptococcus garvieae* sp. nov. and *Streptococcus plantarum* sp. nov. J. Gen. Microbiol..

[3] Kusuda R., Kawai K., Salati F., Banner C.R., Fryer J.L. (1991). *Enterococcus seriolicida* sp. nov., a fish pathogen. Int. J. Syst. Bacteriol..

[4] Eldar A., Ghittino C., Asanta L., Bozzetta E., Goria M., Prearo M., Bercovier H., Bercovier, H (1996). Enterococcus seriolicida is a junior synonym of *Lactococcus garvieae*, a causative agent of septicemia and meningoencephalitis in fish. Curr. Microbiol..

[5] Vendrell D., Balcázar J.L., Ruiz-Zarzuela I., de Blas I., Girones O., Múzquiz J.L. (2006). *Lactococcus* garvieae in fish: A review. Comp. Immunol. Microbiol. Infect. Dis..

[6] Meyburgh C.M., Bragg R.R., Boucher C.E. (2017). *Lactococcus garvieae*: An emerging bacterial pathogen of fish. Dis. Aquat. Organ..

[7] Eldar A., Ghittino C. (1999). *Lactococcus garvieae* and *Streptococcus iniae* infection in rainbow trout (*Oncorhynchus mykiss*): Similar but different diseases. Dis. Aquat. Organ..

[8] Bekker A., Hugo C., Albertyn J., Boucher C.E., Bragg R.R. (2011). Pathogenic Gram-positive cocci in South African rainbow trout, *Oncorhynchus mykiss* (Walbaum). J. Fish Dis..

[9] CONAPESCA Anuario estadístico de acuicultura y pesca 2017. Comisión Nacional de Acuicultura y Pesca, Secretaría de Agricultura Ganadería Desarrollo Rural Pesca y Alimentación. www.conapesca.gob.mx/work/sites/cona/dgppe/2017/ANUARIO_ESTADISTICO_2017.pdf/.

[10] Ortega C., Montes de Oca R., Groman D., Yason C., Nicholson B., Blake S. (2002). Case report: Viral infectious pancreatic necrosis in farmed rainbow trout from Mexico. J. Aquat. Anim. Health..

[11] Castillo M.A., Ortega C., Martínez-Castañeda S., Fajardo-Muñoz R., Valladares-Carranza B., Avendaño-Herrera R., Irgang R., Poblete-Morales M. (2017). First isolation and characterization of *Flavobacterium psychrophilum* from diseased rainbow trout (*Oncorhynchus mykiss*) farmed in Mexico. Bull. Eur. Assoc. Fish Pathol..

[12] Castrejón-Nájera J., Ortega C., Fajardo R., Irgang R., Tapia-Cammas D., Poblete-Morales M., Avendaño-Herrera R. (2018). Isolation characterization, virulence potential of *Weissella ceti* responsible for weissellosis outbreak in rainbow trout (*Oncorhynchus mykiss*) cultured in Mexico. Transbound. Emerg. Dis..

[13] Diario Oficial de la Federación (2018). Secretaria de Gobernación, México. ACUERDO mediante el cual se dan a conocer en los Estados Unidos Mexicanos las enfermedades y plagas exóticas y endémicas de notificación obligatoria de los animales terrestres y acuáticos. https://dof.gob.mx/nota_detalle.php?codigo=5545304&fecha=29/11/2018.

[14] Ravelo C., Magariños B., Romalde J.L., Toranzo A.E. (2001). Conventional versus miniaturized systems for the phenotypic characterization of *Lactococcus garvieae*. Bull. Assoc. Fish Pathol..

[15] Vela A.I., Vázquez J., Gibello A., Blanco M.M., Moreno M.A., Liébana P., Albendea C., Alcalá B., Mendez A., Domínguez L. (2000). Phenotypic and genetic characterization of *Lactococcus garvieae* isolates in Spain from lactococcosis outbreak and comparison with isolates of other countries and sources. J. Clin. Microbiol..

[16] Ortega C., Valladares B., Arguedas D., Vega F., Montes de Oca R., Murray A.G. (2016). Distribution of infectious pancreatic necrosis virus (IPNV) based on surveillance programs in freshwater trout farms of Mexico. J. Aquat. Anim. Health..

[17] Valdebenito S., Avendaño-Herrera R. (2009). Phenotypic, serological and genetic characterization of *Flavobacterium psychrophilum* strains isolated from salmonids in Chile. J. Fish Dis..

[18] MacFaddin J.F. (2000). Biochemical Tests for Identification of Medical Bacteria.

[19] Fox J.G., Yan L.L., Dewhirst F.E., Paster B.J., Shames B., Murpy J.C., Hayward A., Belcher J.C., Mendes E.N. (1995). *Helicobacter bilis* sp. nov., a novel *Helicobacter* species isolated from bile, livers, and intestines of aged, inbred mice. J. Clin. Microbiol..

[20] Lane D.J., Stackebrandt E., Goodfellow M. (1991). 16S/23S rRNA Sequencing in Nucleic Acid Techniques in Bacterial Systematics.

[21] Tamura K., Peterson D., Peterson N., Stecher G., Nei M., Kumar S. (2011). MEGA5: Molecular evolutionary genetics analysis using maximum likelihood, evolutionary distance, and maximum parsimony methods. Mol. Biol. Evol..

[22] Kimura M. (1980). A simple method for estimating evolutionary rates of base substitutions through comparative studies of nucleotide sequences. J. Mol. Evol..

[23] Romalde J.L., Read M.M. (2005). Application of DNA Fingerprinting Techniques to the Study of Fish and Shellfish Pathogens. Trends in DNA Fingerprinting Research.

[24] Ravelo C., Magariños B., López-Romalde S., Toranzo A.E., Romalde J.L. (2003). Molecular fingerprinting of fish-pathogenic *Lactococcus garvieae* strains by random amplified polymorphic DNA analysis. J. Clin. Microbiol..

[25] Ferrario C., Ricci G., Borgo F., Rollando A., Grazia M.F. (2012). Genetic investigation within *Lactococcus garvieae* revealed two genomic lineages. FEMS Microbiol. Lett..

[26] Mancuso M., Avendaño-Herrera R., Zaccone R., Toranzo A.E., Magariños B. (2007). Evaluation of different DNA-based fingerprinting methods for typing *Photobacterium damselae* spp. piscicida. Biol. Res..

[27] Dice L.R. (1945). Measures of the amount of ecologic association between species. Ecology.

[28] Crosa J.H., Hodges L.L. (1981). Outer membrane proteins induced under conditions of iron limitation in the marine fish pathogen *Vibrio anguillarum* 775. Infec. Immun..

[29] Laemmli U.K. (1970). Cleavage of structural proteins during the assembly of the head of bacteriophage T4. Nature.

[30] Mata A.I., Gibello A., Casamayor A., Blanco M.M., Domínguez L., Fernández-Garayzábal J.F. (2004). Multiplex PCR assay for detection of bacterial pathogens associated with warm-water streptococcosis in fish. Appl. Environ. Microbiol..

[31] Yesiltas M.C., Altinok I., Ozturk R.C. (2019). Determination of virulence associated immunogenic proteins in some of *Lactococcus garvieae* strains. Vet. Res. Forum.

[32] Ooyama T., Hirokawa Y., Minami T., Yasuda H., Nakai T., Endo M., Ruangpan L., Yoshida T. (2002). Cell-surface properties of *Lactococcus garvieae* strains and their immunogenicity in the yellowtail *Seriola quinqueradiata*. Dis. Aquat. Organ..

[33] Altun S., Adiloglu A., Kubilay A., Diler O., Delibas N., Sutcu R. (2007). Immunogenic and antigenic profiles of nine *Lactococcus garvieae* strains from different rainbow trout farms. Isr. J. Aquacult Bamid..

[34] Nelson M.C., Varney J.S., Welch T.J., Graf J. (2016). Draft genome sequence of *Lactococcus garvieae* strain PAQ102015-99, an outbreak strain isolated from a commercial trout farm in the Northwestern United States. Genome Announc..

[35] Bercovier H., Ghittino C., Eldar A., Gudding R., Lillehaug A., Midtlyng P.J., Brown F. (1997). Immunization with Bacterial Antigens: Infections with Streptococci and Related Organisms. Fish Vaccinology, Developments in Biological Standardization.

